# Developing a Nationwide Infrastructure for Therapeutic Drug Monitoring of Targeted Oral Anticancer Drugs: The ON-TARGET Study Protocol

**DOI:** 10.3390/cancers13246281

**Published:** 2021-12-14

**Authors:** Anna M. Mc Laughlin, Eduard Schmulenson, Olga Teplytska, Sebastian Zimmermann, Patrick Opitz, Stefanie L. Groenland, Alwin D. R. Huitema, Neeltje Steeghs, Lothar Müller, Stefan Fuxius, Gerald Illerhaus, Markus Joerger, Frank Mayer, Uwe Fuhr, Stefan Holdenrieder, Georg Hempel, Oliver Scherf-Clavel, Ulrich Jaehde, Charlotte Kloft, for the ON-TARGET Study Consortium

**Affiliations:** 1 Department of Clinical Pharmacy and Biochemistry, Institute of Pharmacy, Freie Universität Berlin, 12169 Berlin, Germany; anna.mclaughlin@fu-berlin.de; 2 Graduate Research Training Program PharMetrX, 12169 Berlin, Germany; 3 Department of Clinical Pharmacy, Institute of Pharmacy, University of Bonn, 53121 Bonn, Germany; e.schmulenson@uni-bonn.de (E.S.); o.teplytska@uni-bonn.de (O.T.); u.jaehde@uni-bonn.de (U.J.); 4 Institute of Pharmacy and Food Chemistry, Julius-Maximilians-Universität Würzburg, 97074 Würzburg, Germany; Sebastian.zimmermann@uni-wuerzburg.de (S.Z.); oliver.scherf-clavel@uni-wuerzburg.de (O.S.-C.); 5 Department of Pharmaceutical and Medical Chemistry, Clinical Pharmacy, University of Muenster, 48149 Muenster, Germany; patrick.opitz@uni-muenster.de (P.O.); georg.hempel@uni-muenster.de (G.H.); 6 The Netherlands Cancer Institute, Department of Clinical Pharmacology, Division of Medical Oncology, 1066 CX Amsterdam, The Netherlands; s.groenland@nki.nl (S.L.G.); n.steeghs@nki.nl (N.S.); 7 The Netherlands Cancer Institute, Department of Pharmacy & Pharmacology, 1066 CX Amsterdam, The Netherlands; a.huitema@nki.nl; 8 Department of Clinical Pharmacy, University Medical Center, Utrecht University, 3584 CX Utrecht, The Netherlands; 9 Department of Pharmacology, Princess Máxima Center for Pediatric Oncology, 3584 CX Utrecht, The Netherlands; 10 Onkologie UnterEms, 26789 Leer, Germany; lothar.mueller@onkologie-leer.de; 11 Onkologische Schwerpunktpraxis Heidelberg, 69115 Heidelberg, Germany; stefanfuxius@gmx.de; 12 Klinik für Hämatologie, Onkologie, und Palliativmedizin, Klinikum Stuttgart, 70174 Stuttgart, Germany; g.illerhaus@klinikum-stuttgart.de; 13 Cantonal Hospital St. Gallen, Department of Medical Oncology & Hematology, CH-9000 St. Gallen, Switzerland; Markus.Joerger@kssg.ch; 14 Praxis und Tagesklinik, Prof. Dr. Helmut Oettle und Prof. Dr. Dr. Frank Mayer, 88045 Friedrichshafen, Germany; empfang@onkologie-fn.de; 15 Department I of Pharmacology, Faculty of Medicine and University Hospital Cologne, Center for Pharmacology, University of Cologne, 50923 Cologne, Germany; uwe.fuhr@uk-koeln.de; 16 German Heart Center Munich, Technical University Munich, Institute of Laboratory Medicine, 80636 Munich, Germany; holdenrieder@dhm.mhn.de

**Keywords:** therapeutic drug monitoring, oral anticancer drugs, renal cell carcinoma, volumetric absorptive microsampling

## Abstract

**Simple Summary:**

Relationships between drug concentrations in blood and efficacy and/or toxicity have been reported for up to 80% of oral anticancer drugs (OADs). Most OADs exhibit highly variable drug concentrations at the approved dose. This may result in a significant proportion of patients with suboptimal drug concentrations. Therapeutic Drug Monitoring (TDM), which is dose optimization based on measured drug concentrations, can be used to personalize drug dosing with the overall goal to improve the benefit-risk ratio of anticancer drug treatment. The ON-TARGET study aims to investigate the feasibility of TDM in patients receiving either axitinib or cabozantinib for the treatment of renal-cell carcinoma with the main objective to improve severe tyrosine kinase inhibitor associated toxicity. Additionally, the feasibility of volumetric absorptive microsampling (VAMS), a novel minimally invasive and easy to handle blood sampling technique, for TDM sample collection is investigated.

**Abstract:**

Exposure-efficacy and/or exposure-toxicity relationships have been identified for up to 80% of oral anticancer drugs (OADs). Usually, OADs are administered at fixed doses despite their high interindividual pharmacokinetic variability resulting in large differences in drug exposure. Consequently, a substantial proportion of patients receive a suboptimal dose. Therapeutic Drug Monitoring (TDM), i.e., dosing based on measured drug concentrations, may be used to improve treatment outcomes. The prospective, multicenter, non-interventional ON-TARGET study (DRKS00025325) aims to investigate the potential of routine TDM to reduce adverse drug reactions in renal cell carcinoma patients receiving axitinib or cabozantinib. Furthermore, the feasibility of using volumetric absorptive microsampling (VAMS), a minimally invasive and easy to handle blood sampling technique, for sample collection is examined. During routine visits, blood samples are collected and sent to bioanalytical laboratories. Venous and VAMS blood samples are collected in the first study phase to facilitate home-based capillary blood sampling in the second study phase. Within one week, the drug plasma concentrations are measured, interpreted, and reported back to the physician. Patients report their drug intake and toxicity using PRO-CTCAE-based questionnaires in dedicated diaries. Ultimately, the ON-TARGET study aims to develop a nationwide infrastructure for TDM for oral anticancer drugs.

## 1. Introduction

The development of targeted oral anticancer drugs has significantly improved not only the number of treatment options, but also the prognosis for many tumor entities. Over the last decade, the route of administration has changed from intravenous to oral. This comes with several advantages, such as reduced patient burden due to venipuncture as well as reduction of healthcare costs and a higher flexibility for the patients, as they have to visit their treatment center less often. However, there are also disadvantages: oral anticancer drugs are often administered in complex dosing regimens that are now under the responsibility of the patient which makes the correct intake challenging. Moreover, as patients see their treating physician less often, potential problems such as frequent or severe toxicity or non-adherence might not be identified early on. The majority of targeted oral anticancer drugs displays high pharmacokinetic variability [1,2,3] which results in considerable interindividual differences in the observed drug concentrations after intake of the same dose. Even though exposure-response or exposure-toxicity relationships have been identified for up to 80% of the compounds [3], dosing still mostly follows a “one-dose-fits-all” approach. This fixed dosing results in a significant risk for patients to receive suboptimal (i.e., too high or too low) doses [3,4,5]. Up to 30% of patients with subtherapeutic concentrations have been reported to be at risk for treatment failure while approximately 15% of patients with supratherapeutic concentrations have been reported to be at risk for increased toxicity leading to treatment interruptions or even treatment discontinuation [4]. To prevent therapeutic failure, selecting optimal individual drug doses is crucial.

For drugs with an established exposure-response and/or a exposure-toxicity relationship, measuring drug concentrations in plasma as part of a Therapeutic Drug Monitoring (TDM) framework can be used to detect subtherapeutic concentrations or a high risk for severe or frequent adverse drug reactions due to supratherapeutic concentrations early on [6]. TDM is increasingly being proposed to select optimal individual doses of oral anticancer drugs [7,8,9,10,11,12,13,14,15,16,17]. However, this is usually applied to few patients only, e.g., in the context of clinical trials [3,5] or when they experience particular clinical issues. In ‘traditional’ TDM, drug concentrations are quantified in venous plasma samples [6], requiring the patient to come to the treatment center and undergo venipuncture. As this procedure is time-intensive for both the patient and the treatment team, and venipunctures can be painful, alternative blood sampling methods are increasingly investigated. As a minimally invasive approach, volumetric absorptive microsampling (VAMS), an enhanced development of dried blood spot sampling, allows collecting a small defined volume (10–30 μL) of capillary blood from the patient’s fingertip [18] which is subsequently air-dried and sent to the analytical laboratory using normal post. In contrast to venous blood, using dried capillary blood as matrix for TDM has several advantages: (i) the samples can be collected in a home-based setting [19], (ii) the collected blood samples do not have to be cooled or further processed [19,20], and therefore (iii) the samples can be shipped to the laboratory via normal post [19,20]. The main advantages of VAMS over dried blood spots sampling are the collection of defined blood volumes (10–30 μL), its independence of hematocrit values [18], and its easy handling. The use of VAMS as an alternative to venous blood sampling has already been successfully applied for several compounds such as anti-epileptic drugs [21,22,23,24].

We previously identified a lack of experience and a missing infrastructure as an obstacle to the implementation of TDM for oral anticancer drugs as part of clinical routine [3]. Thus, the observational ON-TARGET study aims to investigate the potential of using routine TDM in clinical practice for reducing the severity and frequency of adverse drug reactions experienced during oral anticancer drug therapy. In addition to the Common Terminology Criteria for Adverse Events (CTCAE) [25], the patients’ perspective of their symptom burden will be assessed in this study by using the Patient-Reported Outcomes version of the CTCAE (PRO-CTCAE) [26] as the CTCAE grading often underestimates the severity and frequency of treatment-related toxicity experienced by patients [27,28,29]. This will further allow exploring the association between drug concentrations and toxicity. Moreover, the study aims to investigate the feasibility, practicability, patient compliance, and acceptance of using home-based VAMS capillary blood sampling as an alternative to venous blood sampling. Ultimately, the ON-TARGET study’s goal is to develop a nationwide infrastructure for routine TDM for oral anticancer drugs.

## 2. Materials and Methods

The ON-TARGET study (short for: Non-interventional study on Therapeutic Drug Monitoring (TDM) in patients with renal cell carcinoma and on the feasibility of using Volumetric Absorptive Microsampling (VAMS) for sample collection) is a prospective, multicenter, non-interventional study (DRKS00025325). Both hospitals and outpatient oncology practices which have implemented TDM as part of their clinical routine can participate as clinical study centers. Patient treatment and prescription of anticancer drugs is at the physician’s discretion and independent from a potential inclusion into the ON-TARGET study. Physicians may propose participation in the ON-TARGET study to (i) patients who experience, or are at high risk of experiencing, frequent and/or severe adverse drug reactions, or (ii) patients for whom TDM could be useful for other reasons (e.g., in the context of suspected non-adherence). The duration of the study participation is at the discretion of the patient and the treating physician.

### 2.1. Inclusion and Exclusion Criteria

Renal cell carcinoma patients at any disease stage receiving either axitinib or cabozantinib in an in-patient or outpatient setting can be included in the study upon provision of their written informed consent. To be eligible for inclusion, patients need to have full legal capacity and be physically, mentally, and based on their verbal skills capable of completing patient questionnaires in German language without the help from a third party. In line with the non-interventional character and to cover a real-world population, patients may be included in the study regardless of their time of treatment start with axitinib or cabozantinib, i.e., both patients already receiving axitinib or cabozantinib and patients starting to receive axitinib or cabozantinib at the time of study inclusion are eligible. In line with the study’s ‘real-world’ setting, patients can be included regardless of their concomitant diseases or co-medication. Furthermore, there is no pre-specified ratio of male/female patients; according to the national cancer database of the Robert Koch Institute in Germany, the 1-year prevalence rate is approximately 1:1.7, which we would also expect to see in our study [30].

### 2.2. Primary Objective

The primary study objective is the prospective evaluation of the potential of routine TDM to reduce the frequency and severity of adverse drug reactions in comparison with the frequency and severity of adverse drug reactions observed in historical controls in renal cell carcinoma patients receiving axitinib or cabozantinib.

### 2.3. Secondary Objectives

Secondary objectives of the study are to identify patient subgroup(s) that experience particularly severe and/or frequent adverse drug reactions. Considering the reported exposure-toxicity relationship for both axitinib [31] and cabozantinib [32], potential plasma concentration thresholds for acceptable toxicity will be explored. Moreover, the feasibility and practicability of using VAMS capillary blood instead of venous blood as matrix for TDM will be investigated.

### 2.4. Study Design

#### 2.4.1. Overview

Patients receiving axitinib or cabozantinib and eligible for inclusion in the ON-TARGET study are approached by their treating physician and informed about the study objectives, the methods applied, and potential risks and benefits associated with the study participation. Patients who are willing to participate must give their written informed consent.

Once a patient has been included in the study, their demographic information, relevant medical history, concomitant diseases, comedication and toxicity are recorded in a dedicated electronic case reporting form (eCRF) during the baseline visit. In addition, they receive one or more patient diary/diaries, depending on the date of the next appointment as each diary covers one month. In these diaries, patients record their daily drug intake along with any optional notes regarding their study treatment and experienced adverse drug reactions. Furthermore, patients complete one PRO-CTCAE questionnaire (see Section 2.4.5) per diary. At the end of each month, patients send their completed diaries to the ON-TARGET coordinating site.

**Figure 1:**
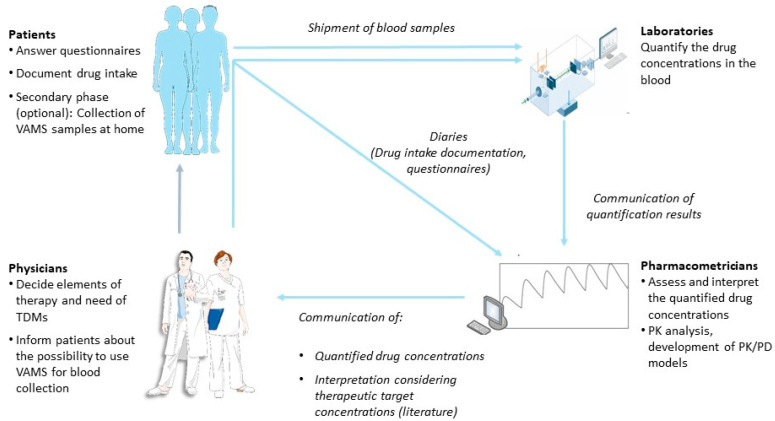
Overview of the ON-TARGET study framework. *Abbreviations*: PD: pharmacodynamics; PK: pharmacokinetics; TDM: therapeutic drug monitoring; VAMS: volumetric absorptive microsampling.

Patients are asked for up to two TDM samples, depending on the study phase (more detailed information in Section 2.4.2 and Section 2.4.3), obtained at a random time during the dosing interval at each study visit. In line with the non-interventional study design, which only allows for blood samples to be obtained as part of standard care, the samples are collected at a single time during the patient’s visit at a treatment center at which TDM is part of clinical routine. Both the last time of drug intake and the time of blood sample collection are documented to derive the time of sample collection within the dosing interval (more detailed information on the evaluation and interpretation is provided in Section 2.4.5). Shipment of VAMS samples occur within 24 h–48 h of sample collection. At the bioanalytical laboratories, concentrations of axitinib or cabozantinib are quantified using methods validated according to the current EMA guidelines [33] and communicated to the pharmacometrics team. The drug concentrations are evaluated, interpreted, and reported back to the treating physician within one week. In line with the non-interventional character of the study, no formal dose recommendations can be made. Drug concentrations are additionally used for evaluating and updating the pharmacokinetic/pharmacodynamic (PK/PD) models. Additional information on cancer disease and any potential change in concomitant diseases, comedication and toxicity is collected. Moreover, exploratory exposure-toxicity analyses for the efficacy biomarkers soluble vascular endothelial growth factors 2 and 3 (sVEGFR2 and sVEGFR3, respectively) [34,35] will be performed. A graphical overview of the study framework is shown in Figure 1.

#### 2.4.2. First Study Phase

In the first study phase, patients are asked to provide both a venous blood sample and a capillary blood sample during their routine visits. For the venous TDM sample, a small volume of approximately 2.7 mL will be separated from the venous blood collected for routine clinical chemistry parameter checks during routine visits. The TDM samples are centrifuged and the plasma aliquoted in 0.5 mL samples for the TDM and biomarker analyses. The capillary TDM samples are collected via volumetric absorptive microsampling using the CE-certified MITRA-VAMS^TM^ (Neoteryx, Torrance, CA, USA) sampling devices. In total, 40 sample pairs are needed to calculate a drug specific conversion factor (capillary blood → venous plasma). This conversion factor is needed for the second study phase, in which home-based capillary blood sampling will be possible (see Section 2.4.3). Assuming that one sample pair will be collected from each patient included in the first study phase, 40 patients will be needed to complete this study phase (see Section 2.5 Statistics). While the first capillary blood sample in the first study phase is always collected by a study nurse, patients will be asked to collect the following capillary blood samples at the treatment center and under the guidance of the study nurses in preparation for the second study phase. This allows ascertaining the correct use of the MITRA-VAMS^TM^ devices and to establish patients’ confidence in using the capillary blood sampling technique.

#### 2.4.3. Second Study Phase

Both patients who already participated in the first study phase and newly recruited patients can participate in the second study phase. Once drug specific conversion factors have been determined, in line with the real-world design of the study, participants will be able to choose between three TDM blood sampling options: (i) venous blood sampling at the treatment center, (ii) capillary blood sampling at the treatment center, and (iii) capillary blood sampling at home. In option (iii), patients send their capillary blood samples to the bioanalytical laboratories via normal post. After quantification of the drug concentrations in capillary blood, the corresponding drug concentrations in venous plasma are derived by applying the conversion factors determined in the first study phase. To assess the practicability of home-based capillary blood sampling, patients will be asked to complete a short questionnaire on their home-based blood sampling experience, e.g., if they experienced any problems, if they would be willing to use home-based capillary blood sampling again, and which blood sampling technique they would prefer in the future. The home-based collection of capillary blood will allow for samples to be sent to the bioanalytical laboratories in advance of patients’ next control visits, allowing the interpreted results to be ready for discussion at the time of the patients’ next visits.

#### 2.4.4. Toxicity Assessment

PRO-CTCAE questionnaires are integrated into the patients’ diaries and are asked to be completed once per month to assess the patients’ symptom burden. The PRO-CTCAE questionnaire used in this study was developed using the linguistically validated German PRO-CTCAE item library [36,37]. A total of 27 questions regarding the severity, frequency, presence or absence, and interference of adverse drug reactions categorized as “very common” or “common” in patients treated with either axitinib or cabozantinib [38,39] were selected for the questionnaire. Furthermore, patients can mention additional adverse drug reactions which are not specifically asked for in the 27 questions. Simultaneously, the patients’ symptom burden is graded using CTCAE version 5.0 [25] once per patient visit.

#### 2.4.5. TDM Result Report

**Figure 2:**
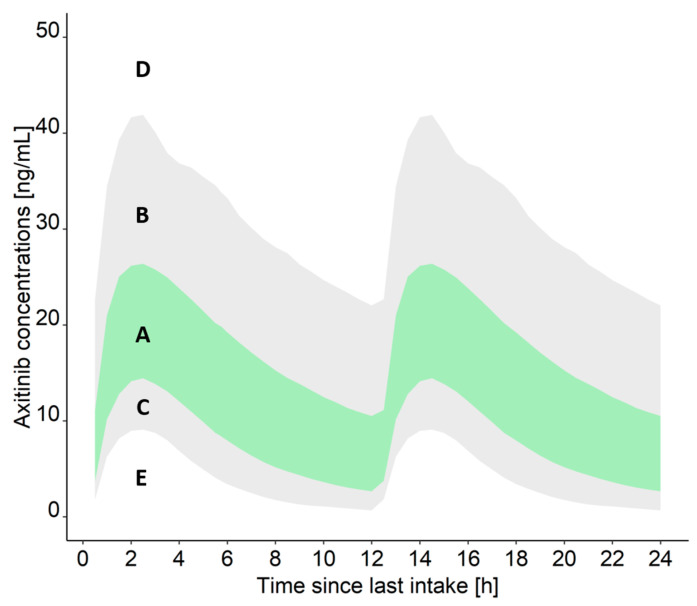
Stochastic simulations for an exemplary axitinib concentration-time profile. Green area: 50% prediction interval (25th–75th percentile), grey area: 90% prediction interval (5th–95th percentile). Letters (A–E) are explained in Section 2.4.5 in the main text.

Date and time of blood sample collection as well as date and time of last drug intake are documented for each patient. In absence of concentration thresholds which should not be exceeded for acceptable toxicity, the measured plasma concentrations are evaluated with respect to the expected concentrations at the time of blood collection after the last dose considering a patient’s individual covariates: For this, previously published nonlinear mixed-effects pharmacokinetic models of axitinib [40] and cabozantinib [41] are used to simulate concentration-time profiles using the patient’s dosing regimen and significant covariates (age, sex, body weight [40,41]) as model input. Stochastic simulations (*n* = 1000) are performed and the 90% prediction intervals (PI) as well as the interquartile range (IQR) together with the measured drug concentration are represented graphically. Depending on the individual drug concentration with respect to the 90% PI and the IQR, a dedicated TDM result summary will be added to the report (Figure 2):

- **Measured drug concentration is within the IQR** (A): The measured concentration is in the *x*th (25th–75th) percentile of the concentrations that can be expected for axitinib/cabozantinib, considering the dosing regimen and relevant covariates of the patient. Thus, the risk of adverse drug reactions is low at the current dosing regimen.
- **Measured drug concentration is within the 90% PI but higher than the IQR** (B)**:** The measured concentration is in the *x*th (76th–95th) percentile of the concentrations that can be expected for axitinib/cabozantinib, considering the dosing regimen and relevant covariates of the patient. Due to the elevated concentrations, the patient should be carefully monitored to detect severe adverse drug reactions early on.
- **Measured drug concentration is within the 90% PI but lower than the IQR** (C)**:** The measured concentration is in the *x*th (5th–24th) percentile of the concentrations that can be expected for axitinib/cabozantinib, considering the dosing regimen and relevant covariates of the patient. Thus, the risk of adverse drug reactions is low at the current dosing regimen.
- **Measured drug concentration is higher than the 90% PI** (D)**:** The measured concentration is in the *x*th (>95th) percentile of the concentrations that can be expected for axitinib/cabozantinib, considering the dosing regimen and relevant covariates of the patient. Thus, a risk of adverse drug reactions is increased at the current dosing regimen and the patient should be carefully monitored.
- **Measured drug concentration is lower than the 90% PI** (E)**:** The measured concentration is in the *x*th (<5th) percentile of the concentrations that can be expected for axitinib/cabozantinib, considering the dosing regimen and relevant covariates of the patient. Thus, according to the result, the patient would not be adequately treated with axitinib/cabozantinib and further investigation for the cause is recommended. Possible causes include a delayed absorption of the drug, non-adherence, drug–drug interactions, or inaccurate documentation of the sample collection time.

### 2.5. Statistics

Based on the exploratory nature of the study, no formal sample size calculations were performed and no formal statistical considerations were made. In general, 30–40 patients per study drug are planned to be included. This number was derived from previous in-house studies, which showed that 30–40 sample pairs (i.e., simultaneous venous and capillary blood sampling) are optimal to identify reliable conversion factors for the calculation of venous drug concentrations from concentrations quantified in capillary blood samples. The usefulness of the TDM will be judged by the participating clinical study centers who are asked to rate the usefulness of the previous TDM on a scale from ‘very helpful’ via ‘somewhat helpful’ and ‘not really helpful’ until ‘not helpful at all’ at each study visit starting from the second visit after the baseline visit. In addition, the study centers are asked if they changed the dosing regimen based on the last TDM result and if yes, in which way (e.g., dose increase or decrease and increase or decrease of the dosing interval). If the dosing regimen was not changed, the study centers are asked to indicate why (e.g., the measured concentration was in the desired range, adverse drug reaction or other). At the end of a patient’s study participation, the study centers are asked to rate the overall usefulness of the TDM for the patient’s treatment. In the second study phase, the proportion of not adequately filled MITRA-VAMS^TM^ devices will be assessed and evaluated. In addition, the duration of shipment and the proportion of lost home-collected VAMS samples will be recorded.

### 2.6. Ethics Considerations, Administration, Data Collection, and Data Protection

The ON-TARGET study (registered in the German Clinical Trials Register (DRKS), https://drks.de (accessed on 7 December 2021), DRKS-ID: DRKS00025325) is conducted in line with the principles of the Declaration of Helsinki. It is currently recruiting at five German study centers who received positive votes by the Ethics committees of the Landesärztekammer Baden-Württemberg (protocol code B-F-2020-159), and the Ärztekammer Niedersachsen (protocol code 159/20). Additional centers can join the consortium and, depending on their location, might require further votes from their respective responsible Ethics committees. Written informed consent is obtained from all patients before study inclusion. A list of collected data and the time points of data collection are shown in Table 1. All information (e.g., data collected during routine visits, TDM results, and patient diaries, including the PRO-CTCAE questionnaire) are captured and stored in an eCRF database. This database is hosted by the commercial cloud computing service provider ShareCRF (Madrid, Spain) who fully adheres to the European General Data Protection Regulation (GDPR) requirements.

## 3. Discussion

Even though exposure-response or exposure-toxicity relationships have been identified for most oral anticancer drugs and the pharmacokinetic variability is often high, individualized dosing is still not routinely performed in oral tumor therapy. Due to the high interindividual PK variability in connection with exposure-response/exposure-toxicity relationships identified for most targeted oral anticancer drugs [3], this one-dose-fits-all approach results in up to 45% of patients receiving a suboptimal dose [4]. Leveraging TDM to facilitate individualized dosing could be a solution to this issue and has so far proven feasible for several targeted oral anticancer drugs (such as abiraterone, everolimus, imatinib, pazopanib, sunitinib, and tamoxifen) in prospective studies [6,42,43]. Despite the evidence for its benefit, TDM is rarely implemented in routine clinical care. There are several possible explanations for this phenomenon: first, to the best of our knowledge, no randomized, prospective clinical study investigating the effect of routine TDM on efficacy and/or safety of oral anticancer drugs has been performed so far [3], which, regardless of the evidence generated in non-randomized studies, might prevent TDM from being recommended in treatment guidelines. Second, at first glance, performing TDM in routine care seems expensive. However, considering the direct and indirect costs of treating patients at suboptimal doses, e.g., the costs resulting from treatment progression in underdosed patients and the costs of treating toxicity in overdosed patients, TDM has already been found to be cost-effective for two targeted oral anticancer drugs (tamoxifen [44] and imatinib [44,45]). Third, ‘traditional’ TDM requires patients to come to their treatment center for venous blood sample collection. This is mostly done within the scope of a routine control visit and usually, the TDM results will only be discussed at the patient’s next control visit. This is time-intensive for both the treatment team and the patient, and it delays the implementation of potentially relevant treatment changes based on the TDM result.

Home-based capillary blood sampling using VAMS offers the opportunity to collect and analyze the blood samples between control visits and to provide the results ready for discussion at the patient’s next check-up. Considering this and the other advantages outlined above, VAMS seems to be a promising tool to significantly improve and simplify routine TDM. However, despite its high potential, VAMS is still a relatively new blood collection method and its feasibility, both from a bioanalytical and a patient acceptance point of view, needs to be further investigated. VAMS is more expensive compared to the more traditional dried blood spot (DBS) technique on filter paper or protein saver cards. However, it has the advantage of using a hematocrit-independent sampling volume (20 µL in our case). On DBS cards, the sampled liquid blood spreads on the DBS card according to the blood viscosity which is related to the hematocrit. Thus, the spot thickness and the subsequently sampled dried spot volume by excision is hematocrit dependent and needs to be corrected. To assess differences between capillary dried blood sampled via the VAMS technique and venous plasma samples, a substance specific correction factor needs to be assessed in vivo in a suitable number of patients. This is due to the partition between cellular blood constituents and plasma as well as the different composition of capillary vs. venous blood. Especially enrichment in or exclusion of a drug from cellular components might lead to a hematocrit dependent correction factor, even if the sampled blood volume is hematocrit independent. A hematocrit dependent correction factor could be addressed by using non-destructive hematocrit determination methods on the VAMS sampler, e.g., near infrared spectroscopy (NIR) as previously used for DBS [46].

There is a growing number of publications about the successful use of VAMS for TDM purposes and to the best of our knowledge, there is no general limitation to the type of drug or molecular mass. Methods have been described for anticancer drugs [47], opioids [48], anti-epileptic drugs [21,49,50], anabolic steroids [51], and monoclonal antibodies [52,53]. Especially in the case of oral anti-cancer drugs, a very recent method paper evaluated the similarity between liquid venous blood and VAMS (sampled from liquid venous blood), indicating that VAMS samples corresponded well to a liquid sample [54]. This underlines the feasibility and appropriateness of using VAMS for cabozantinib and axitinib, especially considering the poor performance for this substance class using DBS [55]. Still, other reports mention problems with recovery, bias, and precision [56,57].

A more detailed review of the methodology as well as its advantages and challenges is beyond the scope of this protocol paper and provided elsewhere [18,58]. A detailed discussion and practical guidelines on method validation can be found in [59,60]. Besides the methodological questions, the practicability of using VAMS for home-based routine TDM sample collection has not yet been thoroughly investigated. Thus, the ON-TARGET study will investigate not only the feasibility of using capillary blood as matrix for targeted oral anticancer drug quantification, but also the patient acceptance of using capillary blood collected at home instead of venous blood collected at the treatment center as TDM samples.

There are several challenges associated with the successful implementation of a TDM process: First, in many situations it is obvious that attaining a certain target concentration is needed to achieve the optimal effect. However, in most cases there is no formal proof that performing a TDM would be beneficial, which often prevents it from being recommended in treatment guidelines. An important reason for this is that prospective randomized controlled studies would have to be performed to provide this proof, and funding for such studies is rarely available. Second, considering the often-missing clear guidelines for when to perform TDM, the added value of a TDM in certain situations, e.g., in case of high toxicity or suspected non-adherence, has to be recognized individually by the treating physician. Third, the blood samples for drug concentration quantification have to be obtained and processed correctly. Sufficient blood volumes must be collected, which can be challenging especially for the capillary blood sample. Fourth, the blood samples have to be shipped to the bioanalysis centers and be analyzed within a short time frame. Fifth, efficient and sensitive bioanalysis methods have to be available. Finally, the evaluation and interpretation of the TDM results has to be quick, concise, and useful. In many cases, no PK target for efficacy and/or toxicity is available yet [2,3,61]. For these drugs, the mean/median exposure identified from other studies [2,61] in conjunction with the expected individual expected exposure based on patient’s covariates could be used until a PK target is available.

The ON-TARGET study focuses on the potential of routine TDM to reduce the frequency and severity of adverse drug reactions experienced during oral tumor therapy. There are several additional possible endpoints with regards to the potential of TDM that could and should be addressed in dedicated follow-up studies: first, considering that approximately 30% of patients are underdosed at the recommended standard dose, efficacy is a highly relevant endpoint. Investigating the potential of routine TDM to increase efficacy using hard endpoints (such as overall survival) with sufficient power requires large sample sizes and long follow-up times, also as overall cancer prognoses improve with new treatments. A less cost-intensive option is to focus on surrogate endpoints, e.g., the reduction of the frequency of patients with drug exposure below the TDM target, as it is done in the Dutch DPOG-TDM study [5]. Of note, even though it is often possible to increase drug exposure with cost-neutral interventions [13,14,62,63], using TDM to optimize efficacy will usually also require dose escalations. These dose escalations can lead to the prescription of off-label doses, which should be considered and accounted for early on when selecting an appropriate study design (e.g., interventional, non-interventional). Another endpoint of high relevance is the improvement of treatment adherence and the detection of non-adherence. According to recent literature, non-adherence to targeted oral anticancer drugs is frequent [64,65,66,67,68] and a serious risk factor for treatment failure [69]. Routine TDM may allow the early identification of treatment non-adherence as indicated by drug concentrations significantly lower than expected for a given patient and dosing regimen. For example, in a recent prospective study applying a pharmacokinetic threshold for non-adherence, one in six breast cancer patients receiving tamoxifen was found to be non-adherent [70]. Given the prevalence of non-adherence in targeted oral anticancer drug treatment of 29–59% [67], the potential of routine TDM to identify non-adherence early on should be further investigated in prospective studies.

When developing the protocol for a study to investigate the potential of routine TDM, not only the endpoints but also the degree of the intervention must be considered. In line with its non-interventional character, the ON-TARGET study does not allow for dosing recommendations to be made and possible changes to the dosing regimen are solely at the discretion of the treating physician. While some regulatory authorities allow concrete dosing recommendations within the framework of a non-interventional study [5], others only allow them as part of an interventional trial. Based on their nature of investigating the effect of a change to the standard of care (‘intervention’), interventional studies come with a significant increase in administrative and financial efforts compared to non-interventional studies. However, there are several advantages associated with the opportunity to provide dosing recommendations, which may justify the additional complexity and costs of an interventional study. While more and more clinicians have expertise in and experience with TDM, dosing recommendations provided by experts in pharmacokinetics/pharmacodynamics and TDM will help in the decision-making process upon receiving TDM results especially in treatment centers in which TDM is implemented as a new element in routine care. In addition, applying pharmacokinetic/pharmacodynamic modeling and simulation as part of a model-informed precision dosing framework allows the integrated analysis of a patient’s TDM result for dose selection, considering multiple sources of information including but not limited to disease and medication history, current comedication, genetic information, organ function, and adherence [71,72,73]. Moreover, using dedicated treatment simulations, several new dosing regimens can be tested in silico before deciding on which changes to implement in vivo.

While the ON-TARGET study is currently in its early stage, it can and is planned to be extended to more compounds and cancer entities. Furthermore, the developed infrastructure can be further leveraged in a future interventional study investigating the potential of model-informed precision dosing for targeted oral anticancer drugs in clinical routine. Applying home-based capillary blood sampling together with model-informed PK/(PD) assessment in clinical routine will provide precise dosing recommendations based on measured drug concentrations before the patient’s next control visit while minimizing the time effort needed from both patients and treatment centers.

## 4. Conclusions

The non-interventional ON-TARGET study investigates the potential of routine TDM to reduce the frequency and severity of adverse drug reactions during targeted oral anticancer drug therapy. An infrastructure of clinical and academic experts in oral tumor therapy, pharmacometrics, bioanalysis of both drug concentrations and biomarkers, and clinical studies has been successfully implemented in Germany. In the next step, the feasibility of home-based capillary blood sampling for TDM sample collection instead of traditional venous blood sampling will be investigated. The ON-TARGET study can be extended to offer routine TDM and, within an interventional setting, model-informed precision dosing, for a large number of oral anticancer drugs.

## Figures and Tables

**Table 1:** Overview of the data collected and the time of collection in the ON-TARGET study.

Data Collection Parameter	Baseline Visit	Follow-Up Visits	Continuous
Vital parameters	X	X	
Information on current tumor therapy (treatment setting, current medication, concomitant treatment)	X	X	
Information on previous tumor therapies	X		
Information on tumor diagnostics	X	X ^1^	
Tumor classification according to TNM	X	X ^1^	
Concomitant diseases	X	X ^2^	
Current supportive medication	X	X ^2^	
Current comedication	X	X ^2^	
Toxicity according to CTCAE 5.0	X	X ^2^	
Symptomatic toxicity according to PRO-CTCAE			X ^3^
Drug intake			X ^4^
ECOG performance status	X	X	
Clinical chemistry parameters	X	X	
If applicable, information on the treatment termination (Date, reason(s))		X	

^1^ Only if tumor staging was performed; ^2^ Only if changes occurred since the last visit; ^3^ Filled in once monthly by the patient. ^4^ Filled in daily by the patient. Abbreviations: CTCAE: Common Terminology Criteria for Adverse Events; PRO: patient-reported outcomes; ECOG: Eastern Cooperative Oncology Group; TNM: tumor (T), nodes (N), metastasis (M).

## Data Availability

No new data were created or analyzed in this study. Data sharing is not applicable to this article.
